# Supplementary figures and images for: Targeted knockdown of DNA methyltransferase 3a (DNMT3a) unlocks dedifferentiation and neurogenic potential in mouse retinal Müller glia
Source: PLoS One. 2025 Dec 17;20(12):e0337891. doi: 10.1371/journal.pone.0337891 (PMC12711019; doi:10.1371/journal.pone.0337891)

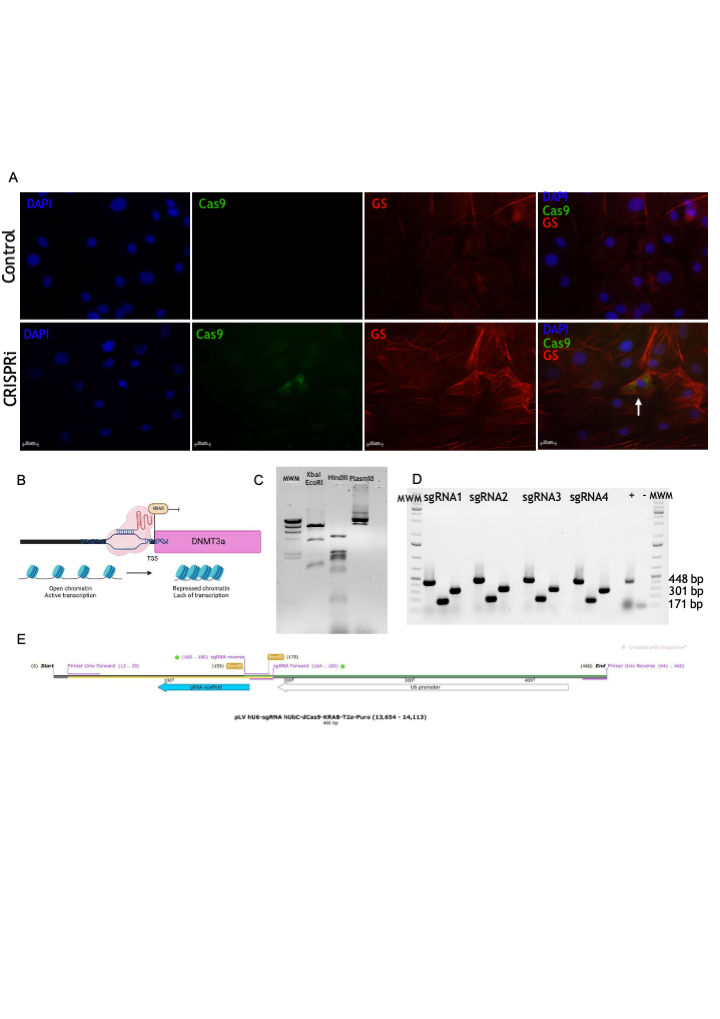

Supplement: S2 Fig — A) Representative immunofluorescence imagen of CTRL and DNMT3a KD groups to validate Müller cell transfection protocol, determined by Cas9 protein (green) for plasmid and glutamine synthetase (GS, red) for Müller cells delimitation. Nuclei were stained with DAPI (blue). Scale bar = 20 µm, 40X. B) Schematic representation of the general mechanism of action of CRISPRi systems. C) Representative agarose gel for plasmid digestions. D) Representative agarose gel for conventional PCR products for guide RNA (sgRNA) ligation into plasmid vector + : positive control to U6 promoter, sgRNA and gRNA scaffold fragment amplification; -: non template control; MWM: molecular weight marker. E) Schematic representation of PCRs amplifications presented in panel D, generated with SnapGene. (TIFF) [file pone.0337891.s002.tiff]
