# Supplementary material for: Targeted knockdown of DNA methyltransferase 3a (DNMT3a) unlocks dedifferentiation and neurogenic potential in mouse retinal Müller glia
Source: PLoS One. 2025 Dec 17;20(12):e0337891. doi: 10.1371/journal.pone.0337891 (PMC12711019; doi:10.1371/journal.pone.0337891)
Supplement: Attachment — Submitted filename: Response to the Reviewers.docx [file pone.0337891.s005.docx]

Response to the Reviewers

Reviewer #1:

To fully contextualize the significance of these findings, the authors should add a comparative table contrasting zebrafish MG regeneration, prior mammalian MG reprogramming strategies (e.g., Ascl1 + HDACi, TF cocktails), and the current DNMT3A-targeting approach, highlighting differences in injury triggers, molecular drivers, epigenetic states, and regenerative outcomes.

For future studies, it would be valuable to discuss the potential of small-molecule DNMT inhibitors, siRNA approaches, or TET inhibitors (e.g., Bobcat339) as orthogonal validation strategies, as well as the importance of direct epigenomic profiling (DNA methylation/5hmC, ATAC-seq) and testing within injury and immune contexts where microglia are known to modulate MG reprogramming.

With these additions, this work will not only stand as a strong mechanistic advance but also serve as a roadmap for translational strategies aimed at inducing regeneration in the mammalian retina.

**We sincerely thank you for your thoughtful and constructive review, which has significantly improved our manuscript. In response to your suggestion, we have now included a comparative table (Table 1).**

**To comply with your suggestions we have included the following paragraph in the Discussion section toghether with several new references:**

***“Dnmt3a* CRISPR-dCas9-mediated downregulation may represent a promising strategy to prolong the transient progenitor state of MG and enhance their neurogenic potential and retinal regenerative capacity in mammals. Importantly, this approach could complement several reprogramming strategies previously applied in both regenerative and non-regenerative species (Table 1). Orthogonal validation methods, such as small-molecule epigenetic modulators (73), siRNAs (27), or CRISPR-CasRx (72), would be invaluable to reinforce the robustness and significance of these findings.**

**For these observations to have a physiological impact, it is essential to translate these studies into an in vivo system, where the interaction of Müller cells with all retinal and immune cells, including microglia, can be properly taken into account. Although complex, this is a feasible task. The CRISPR/Cas system has been successfully adapted to target retinal cells in vivo, either through electroporation (74, 75) or by adeno-associated virus 2 (AAV2)-mediated deliver, an approach that holds greater promise for future clinical applications (76).”**

Minor comment: page 22 (Kiy should be Kit).

**Corrected.**

**Thank you very much for all your suggestions.**

Reviewer #2: 
Major Points for Revision:

1.The title and abstract emphasize "transient inhibition," but the method is a knockdown via transfection. While the effect may not be permanent, "transient" could be misinterpreted. Clarify the duration of the knockdown effect (shown to be ~3-5 days) and justify the terminology or consider rephrasing (e.g., "targeted knockdown").

**We have incorporated your suggestion by revising our title to “Targeted knockdown of DNA methyltransferase 3a (DNMT3a) unlocks Dedifferentiation and Neurogenic Potential in Mouse Retinal Müller Glia”; and by clarifying the duration of the knockdown effect in the text.**

2.A discussion on the potential and challenges of translating this in vitro CRISPRi approach to an in vivo mammalian model is crucial. This should be added to the Discussion.

**Taking into consideration the reviewer´s suggestions we have now enriched our Discussion with the following paragraph:**

**“For these observations to have a physiological impact, it is essential to translate these studies into an *in vivo* system, where the interaction of Müller cells with all retinal and immune cells, including microglia, can be properly taken into account. Although complex, this is a feasible task. The CRISPR/Cas system has been successfully adapted to target retinal cells *in vivo*, either through electroporation (74, 75) or by adeno-associated virus 2 (AAV2)-mediated deliver, an approach that holds greater promise for future clinical applications (76). Although they offer high efficiency and specificity, when compared for example with the available pharmacological DNMT inhibitors, CRISPR/Cas9 gene editing technologies are not without limitations. These include potential off-target effects, variability in editing efficiency across cell types, immune responses against Cas proteins or viral vectors, and challenges in achieving precise spatial and temporal control of gene modulation within the retina to achieve sufficient regeneration. Resolving these issues may open new therapeutic avenues for retinal diseases.”**

3.Several figures (as placeholder text) are referenced but not available for review. The figure legends must be exceptionally clear. Ensure all panels are labeled (A, B, C, etc.), statistical test details (n numbers, exact p-values where possible) are included, and scale bars are visible on the final images.

**We have revised our document to avoid errors. Thank you.**

Specific Comments by Section
1. Title: It is accurate but slightly long.

**We have revised our title to “Targeted knockdown of DNA methyltransferase 3a (DNMT3a) Unlocks dedifferentiation and Neurogenic Potential in Mouse Retinal Müller Glia”**

2. Introduction：Consider adding a sentence explicitly stating the knowledge gap: e.g., "While the role of demethylation has been explored, the specific contribution of de novo methyltransferases like DNMT3a in restricting mammalian MG plasticity remains unclear."

**Thank you very much. We have added this sentence in page 4.**

3. Discussion The co-expression of glial and neuronal markers (Fig 7) needs to be discussed in more depth. What does this mean for the functionality of these cells? Could this be an intermediate state that requires additional cues for full maturation? This is a key point for the field.

**Indeed, this is a key point for the field. However, there is not a lot of information as most of the articles on the area do not address the co-expression of glial and neuronal markers. We wanted to include this figure to start adding to the evidence, convinced that more examples are going to be reported. However, it is still too early for us to fully discuss possible physiological implications of these observations. We have now included in the text our “future direction”. It is an ongoing but promising field.**

**This is the sentence that we have now included in the Discussion section:**

**“Further electrophysiological characterization of βIII-tubulin–expressing cells will be required to confirm their neuronal functionality. Such analyses could also help determine whether, for instance, temporal control of DNMT3a expression might extend the window of neuronal differentiation, thereby leading not only to an increased number of neurons but also to the acquisition of a fully functional neuronal phenotype.”**

As noted above, a paragraph discussing the practical challenges and future possibilities of applying this strategy in vivo (e.g., using AAV-delivered CRISPRi) is necessary. Would transient inhibition be sufficient to stimulate regeneration in a model of retinal degeneration?

**Here is the paragraph that we included:**

**“For these observations to have a physiological impact, it is essential to translate these studies into an *in vivo* system, where the interaction of Müller cells with all retinal and immune cells, including microglia, can be properly taken into account. Although complex, this is a feasible task. The CRISPR/Cas system has been successfully adapted to target retinal cells *in vivo*, either through electroporation (74, 75) or by adeno-associated virus 2 (AAV2)-mediated deliver, an approach that holds greater promise for future clinical applications (76). Although they offer high efficiency and specificity, when compared for example with the available pharmacological DNMT inhibitors, CRISPR/Cas9 gene editing technologies are not without limitations. These include potential off-target effects, variability in editing efficiency across cell types, immune responses against Cas proteins or viral vectors, and challenges in achieving precise spatial and temporal control of gene modulation within the retina to achieve sufficient regeneration. Resolving these issues may open new therapeutic avenues for retinal diseases.”**

4. Materials and Methods：The protocol for obtaining pure MG cultures is standard. However, stating the final percentage of GS-positive cells (e.g., >95%) from an immunofluorescence validation would strengthen the methodology. Please include the efficiency of electroporation/transfection, as determined by Cas9 immunofluorescence. This is important for interpreting the % of cells showing effects.

**This information has been included in the text:**

**“More than 95% of the cells were immunopositive to GS (not shown).”**

**“After puromycin selection more than 90% of the cells were immunopositive to Cas9.”**

Thank you very much for your help.

Reviewer #3:

1. As mentioned in the introduction, DNMT3a, has been identified as a crucial regulator in optic nerve regeneration, and its downregulation has been shown to induce passive demethylation at neurogenic gene loci in different cell types. In addition, a recent review (Zhou et al., 2025; Advancements in Müller Glia Reprogramming: Pioneering Approaches for Retinal Neuron Regeneration) emphasizes that DNA demethylation is crucial for initiating Müller glia reprogramming. These observations raise questions regarding the distinction of this study from previous works. The authors should clarify more explicitly what is novel about their study.

**Indeed, DNMT3 has been identified as a crucial regulator of retinal ganglion cell regeneration and axonal growth. However, no role for this enzyme has yet been described in Müller glia. To explicitly highlight the novelty of our study, we have included the following sentence in the Introduction:**

**“While the role of demethylation has been explored, the specific contribution of *de novo* methyltransferases like DNMT3a in restricting mammalian MG plasticity remains unclear.”**

2. Off-target effects of CRISPRi are only minimally addressed. The authors should point out that the knockdown of DNMT3b and DNMT1 has not been evaluated beyond qPCR.

**We have now precisely specified this in page 6.**

**“Specificity of the effect of the inhibition system on *Dnmt3a* expression was confirmed by RT-qPCR evaluation of the expression of *Dnmt3b* and *Dnmt1* under the same experimental conditions. No significant effects were observed (Fig. 1 B,C), although possible effects of *Dnmt3b* and *Dnmt1* were not evaluated beyond qPCR.”**

3. The results indicate that DNMT3a knockdown induced βIII-tubulin expression in the cells, while glial markers were also retained. This suggests only partial reprogramming of Müller glia cells. The authors should address this as a limitation and discuss possible solutions as future directions.

**Indeed, this is a key point for the field. However, there is not a lot of information as most of the articles on the area do not address the co-expression of glial and neuronal markers. We wanted to include this figure to start adding to the evidence, convinced that more examples are going to be reported. However, it is still too early for us to fully discuss possible physiological implications of these observations. We have now included in the text our “future direction”. It is an ongoing but promising field.**

**This is the sentence that we have now included in the Discussion section:**

**“Further electrophysiological characterization of βIII-tubulin–expressing cells will be required to confirm their neuronal functionality. Such analyses could also help determine whether, for instance, temporal control of DNMT3a expression might extend the window of neuronal differentiation, thereby leading not only to an increased number of neurons but also to the acquisition of a fully functional neuronal phenotype.”**

4. The use of the CRISPRi system for the transient downregulation of DNMT3a is elegant and specific, but technically demanding. The authors should explain clearly the advantages and disadvantages of this system over pharmacological inhibition by small-molecule DNMT inhibitors, which are easier to apply.

5. Although no significant negative impact of transient DNMT3a inhibition by the CRISPRi system on cell viability was observed in vitro, the possible safety concerns should be briefly discussed. These include the possibility of aberrant or incomplete reprogramming, deregulation of additional pathways, uncontrolled proliferation or oncogenic transformation, and instability of epigenetic state. Given the therapeutic aim of this study, the authors should acknowledge the associated risks and highlight that further in vivo safety and functional studies are essential before considering any clinical application. A brief discussion of these points would strengthen the therapeutic relevance of the manuscript.

**Regarding these two comments we have included this new information in the Dicussion section:**

**“For these observations to have a physiological impact, it is essential to translate these studies into an *in vivo* system, where the interaction of Müller cells with all retinal and immune cells, including microglia, can be properly taken into account. Although complex, this is a feasible task. The CRISPR/Cas system has been successfully adapted to target retinal cells *in vivo*, either through electroporation (74, 75) or by adeno-associated virus 2 (AAV2)-mediated deliver, an approach that holds greater promise for future clinical applications (76). Although they offer high efficiency and specificity, when compared for example with the available pharmacological DNMT inhibitors, CRISPR/Cas9 gene editing technologies are not without limitations. These include potential off-target effects, variability in editing efficiency across cell types, immune responses against Cas proteins or viral vectors, and challenges in achieving precise spatial and temporal control of gene modulation within the retina to achieve sufficient regeneration. Resolving these issues may open new therapeutic avenues for retinal diseases.”**

6. The neural differentiation analysis focuses on morphology and marker expression (βIII-tubulin), without functional validation. At minimum, the authors should acknowledge the limitation of relying solely on this early neuronal marker and emphasize that additional differentiation markers or functional assays will be required to confirm neuronal identity and functionality.

**We agree. We have included a statement indicating “Further electrophysiological characterization of βIII-tubulin-expressing cells will be required to confirm neuronal functionality”.**

**Thank you very much for your review.**
