# Supplementary material for: Targeted knockdown of DNA methyltransferase 3a (DNMT3a) unlocks dedifferentiation and neurogenic potential in mouse retinal Müller glia
Source: PLoS One. 2025 Dec 17;20(12):e0337891. doi: 10.1371/journal.pone.0337891 (PMC12711019; doi:10.1371/journal.pone.0337891)
Supplement: Attachment — Submitted filename: Response to the Reviewers 2.docx [file pone.0337891.s006.docx]

Reviewers' Response

**Reviewer #1: The paper is much improved and the inclusion of a table comparing zebrafish and mouse 3a inhibition studies is helpful. I only have a couple of minor corrections in the table: dcas9 should be dCas9 - and explain what CasRx and dCas9 are in the table or text.**

The typo has been corrected and information about CasRx (Cas13d) has been included in the text, thank you.

In line 325 of the Results section, dCas9 is described in the text as a “catalitically inactive Cas9”.

“These sgRNAs were incorporated into CRISPR interference systems (CRISPRi) utilizing a catalytically inactive Cas9 (dCas9) fused to the Krüppel-associated box (KRAB) repressor domain (43) (S1). This configuration enables targeted transient transcriptional repression of *Dnmt3a* expression in transfected cells.”

In line 518 of the Discussion, brief information about CasRx is provided:

“Orthogonal validation methods, such as small-molecule epigenetic modulators (73), siRNAs (27), or the recently characterized and optimized CRISPR protein family Cas13, also known as CRISPR-CasRx (72), would be invaluable to reinforce the robustness and significance of these findings”

**Reviewer #3: The authors have comprehensively responded to the reviewers’ comments, and the manuscript has been substantially improved. However, several aspects would still benefit from further clarification.
1. The novelty of the study relative to previous works on DNMT3A-related reprogramming should be explained more explicitly. What distinguishes the present CRISPRi-based approach from prior demethylation strategies? Is the CRISPRi system preferable to previously published methods, and if so, why?**

**2. The authors should discuss the potential risks associated with partial reprogramming of the Müller glia cells, along with the possible solutions in the future.**

Combining these two questions, we have added this information in the Dsicussion Section:

“CRISPRi systems offer high efficiency and specificity, when compared for example with the available non-specific pharmacological DNMT inhibitors (e.g., 5-azacytidine or RG108) which broadly reduce methylation across the genome and may indirectly affect multiple cellular pathways, often leading to cytotoxicity or limited temporal control. The use of a catalytically inactive Cas9 (dCas9) fused to the KRAB repressor domain enabled precise, locus-specific and transient transcriptional repression of Dnmt3a, simultaneously minimizing off-target disturbances and preserving the necessary temporal reversibility of the epigenetic alteration. In addition, these procedures are scalable and should permit the evaluation of the coordinated regulation of other transcription factors.

However, CRISPR/Cas9 gene editing technologies are not without limitations, and resolving such limitations could overcome some of the potential risks that partial reprogramming of Müller glia carry. For example, incomplete or unstable reprogramming may lead to **cellular heterogeneity**, with subpopulations that co-express glial and neuronal markers but fail to achieve full neuronal functionality. Such intermediate states could result in **aberrant signaling, impaired glial support functions,** or even **uncontrolled proliferation** resembling gliosis or tumor-like growth. Combining **gene-specific epigenetic modulation (e.g., Dnmt3a CRISPRi)** with **controlled activation of pro-neural transcription factors** could promote more complete lineage conversion while preserving retinal architecture.

In addition, if induced epigenetic alterations persist over time, they may lead to **epigenomic instability.** Future studies could employ **inducible CRISPR-based systems** to **r**estrict gene modulation to defined temporal windows, thereby ensuring that regenerative outcomes enhance repair without compromising retinal homeostasis. Such refined control may ultimately provide safer and more effective therapeutic avenues for retinal diseases.”

**3. As mentioned in the previous review round, the assessment of neuronal differentiation relies mainly on the morphology and the expression of βIII-tubulin (an early neuronal marker). To confirm neuronal identity and functionality, the authors should examine the expression of additional markers associated with the later stages of differentiation, like MAP2 and NEFL. Electrophysiological characterization would also be helpful to verify the final maturation of the neurons.**

Yes, we fully agree with you, and we are currently developing that project. We believe that immunological characterization alone is insufficient and would leave important questions unresolved. Therefore, we are conducting an extensive electrophysiological analysis. Müller cells pose certain challenges for patch-clamp recordings, whereas progenitor cells and βIII-tubulin–expressing cells are more readily analyzed and display distinct electrophysiological patterns. Our preliminary observations reveal a considerable degree of heterogeneity in the electrophysiological responses of these cells. As you rightly note, this is a critical issue, and we prefer not to draw premature conclusions at this stage. We have revised the Discussion to clarify this point and hope to be granted the time necessary to fully address this important aspect.

“Further genotypic characterization of βIII-tubulin–expressing cells, through the evaluation of their immunoreactivity to MAP2 or NeuroD1 antibodies, as well as electrophysiological assessment of their intrinsic properties, is required to confirm their neuronal functionality”.

Thank you very much for your review.
